# Metabolic profiling of retinal organoids reveals conserved core metabolites and alterations in glycolytic and amino acid metabolism

**DOI:** 10.1007/s11306-026-02523-4

**Published:** 2026-10-10

**Authors:** Pragati Gupta, Claudia Wiesner, Focke Ziemssen, Jeroen Klevering, Purva Kulkarini, Zohreh Hosseinzadeh

**Affiliations:** 1 Department of Ophthalmology, Radboud University Medical Centre, Nijmegen, Netherlands; 2 University Eye Hospital Leipzig, Leipzig University, Leipzig, Germany; 3 Faculty of Chemistry, Institute of Analytical Chemistry, Leipzig University, Leipzig, Germany; 4 Laboratory of Genome Diagnostics, Department of Human Genetics, Radboud University Medical Centre, Nijmegen, Netherlands

**Keywords:** Retinal organoids, Mouse retina, Metabolomics, GCMS (Gas chromatography-mass spectrometry)

## Abstract

**Introduction:**

Retinal organoids have emerged as powerful models of retinal development and disease; however, the extent to which they recapitulate the metabolic landscape of the native retina remains largely unknown.

**Objectives:**

This study aimed to compare the metabolomic profiles of mouse retina and mouse embryonic stem cell-derived retinal organoids and to evaluate metabolic changes during organoid maturation.

**Methods:**

Untargeted gas chromatography mass spectrometry based metabolomic profiling was performed on intracellular metabolites from mouse retina at postnatal day 3 and mouse retinal organoids (MROs) at differentiation day 28, together with conditioned media collected during organoid maturation.

**Results:**

Across mouse retina and retinal organoids, 166 annotated compounds were included in the analysis . Of these, 148 were shared between the two sample types. Using a bidirectional fold-change threshold, differential analysis showed that mouse retinal organoids preserved a substantial metabolic signature shared with mouse retina. However, selective differences were observed in the abundance of glycolysis-associated metabolites and pentose phosphate intermediates including glucose-6-phosphate and ribulose-5-phosphate, as well as tricarboxylic acid-cycle-associated metabolites including citric acid, fumaric acid and oxalic acid. Pathway enrichment analysis identified significant alterations in alanine, aspartate, and glutamate metabolism. Media analysis further revealed stage-dependent changes in extracellular glutamate and glycine abundance during organoid maturation.

**Conclusion:**

MRO retains major components of retinal metabolic organization while exhibiting features suggestive of incomplete metabolic maturation compared with native retina. These findings provide valuable insight into MRO metabolic fidelity and guide future optimization strategies.

**Supplementary Information:**

The online version contains supplementary material available at https://doi.org/10.1007/s11306-026-02523-4.

## Introduction

The retina is among the most metabolically active tissues in the body, requiring tightly coordinated energy production, redox homeostasis, and biosynthetic capacity to sustain phototransduction, synaptic transmission, and continuous outer segment renewal (Du et al., 2016; Hurley et al., 2015). Photoreceptors rely heavily on aerobic glycolysis, mitochondrial oxidative phosphorylation, and lipid metabolism to meet these demands, while Müller glia and the retinal pigment epithelium provide critical metabolic support through lactate shuttling, antioxidant buffering, and nutrient recycling (Li et al., 2020; Viegas & Neuhauss, 2021).

Direct functional measurements in isolated mouse retina demonstrate that retinal energy metabolism is dynamically regulated by neuronal activity, with flicker stimulation inducing rapid increases in oxygen consumption and metabolic flux, highlighting the tight coupling between retinal function and metabolic demand (Linsenmeier & Dmitriev, 2024). Real-time bioenergetic profiling of dissociated mouse photoreceptors further shows that these cells maintain distinct glycolytic and mitochondrial respiration programs (Yera et al., 2025). Recent studies have highlighted metabolism as a critical determinant of retinal development and function. In the mouse retina, photoreceptors preferentially convert glucose to lactate even under oxygen-rich conditions, while channeling mitochondrial metabolism toward biosynthesis rather than maximal ATP production (Li et al., 2020). Age-dependent remodeling of glycolysis, the tricarboxylic acid (TCA) cycle, amino acid metabolism, and redox pathways have been documented in the mouse retina and retinal pigment epithelium (Tsantilas et al., 2021). Large-scale metabolomic profiling of mouse fetal organs reveals that metabolic pathway composition is organ-specific and developmentally regulated, indicating that metabolic identity is a defining feature of tissue maturation rather than a secondary consequence of differentiation (Li et al., 2025). Consistent with this, early mouse embryogenesis is characterized by extensive metabolic remodeling across glycolysis, the TCA cycle, and lipid metabolism; disruption of these processes impairs developmental progression, demonstrating a causal role for metabolic programming in tissue maturation (Zhao et al., 2021). Importantly, metabolic specialization differs not only across developmental stages but also between rods and cones, retinal regions, and supporting cell types, suggesting that incomplete metabolic patterning may directly limit organoid functionality.

Pluripotent stem cell–derived retinal organoids have emerged as a powerful complementary model system to in vivo mouse studies, offering several experimental advantages for studying retinal development and disease mechanisms (Cowan et al., 2020; Eiraku et al., 2011; Kim et al., 2019). Unlike in vivo mouse models, MROs can be generated in large numbers from a single differentiation batch, enabling parallel experimentation, improved reproducibility, and systematic manipulation of culture conditions that are difficult to achieve in vivo (Kim et al., 2019; La Torre, 2016). This scalability facilitates high-throughput molecular profiling and controlled perturbation studies, while reducing animal usage and experimental variability (Hanna et al., 2022).

At the RNA level, bulk transcriptomics data show that retinal organoids recapitulate key gene expression programs of retinal development, photoreceptor differentiation, and synaptic signalling, but exhibit partial metabolic divergence from native retina (Cui et al., 2020). Most major retinal cell classes are present in organoids, although their molecular identities often correspond to earlier developmental stages compared with native retina, particularly for photoreceptors and metabolically demanding neuronal subtypes (Cowan et al., 2020; Sridhar et al., 2020). Genes associated with energy metabolism, mitochondrial function, lipid biosynthesis, and neurotransmitter cycling are expressed in retinal organoids, indicating partial activation of retinal metabolic pathways at the RNA level (Cui et al., 2020). In retinal organoids, deficiencies in lipid uptake, fatty acid elongation, and β-oxidation have been linked to impaired outer segment development and photoreceptor vulnerability, further emphasizing the need to address metabolic insufficiencies to achieve functional parity with native retina (West et al., 2022). However, transcriptomic and proteomic profiling alone cannot capture actual metabolite abundance or pathway flux and therefore do not directly reflect the functional metabolic state of retinal organoids (Cui et al., 2020). Given that the retina is among the most metabolically demanding tissues in the body, relying heavily on tightly regulated glucose, lipid, and amino acid metabolism to sustain phototransduction and synaptic transmission (Hanna et al., 2022; Viegas & Neuhauss, 2021), indirect molecular readouts may overlook critical metabolic deficiencies affecting organoid maturation. Several studies have developed MROs (Chen et al., 2020; La Torre, 2016; Völkner et al., 2021). Compared with human retinal organoids (~ 200 days), they exhibit a shorter maturation time (~ 28 days) and are more cost-effective. In addition, they can be generated on a large scale in a single batch compared with mouse retina preparation (Chen et al., 2020; La Torre, 2016; Völkner et al., 2021) Therefore, this study uses MROs as an efficient model to support the 3R principles (reduction, replacement, and refinement) and to investigate their metabolic profile at maturity.

In this study, we systematically compare the metabolic landscape of stem cell-derived MROs with that of native mouse retina using untargeted metabolomic profiling and pathway enrichment analysis. By mapping significant differential metabolites to canonical metabolic pathways, we aim to pinpoint candidate metabolic processes that may guide future culture optimization and experimental modulation.

## Materials and methods

### Materials and chemicals

Mouse embryonic stem cells (mESCs; ESD3 line) were cultured using Dulbecco’s Modified Eagle Medium (DMEM), high glucose (Gibco™, Cat# 10564-011), leukemia Inhibitory Factor (LIF; Merck Millipore, Cat# ESG1107), Glasgow’s MEM (GMEM; Gibco™, Cat# 11710-035), and DMEM/F12 Ham (Gibco™, Cat# 10565-018) from Thermo Fisher Scientific (Waltham, MA, USA). Media were supplemented with ESC-qualified fetal bovine serum (FBS; Cat# 10439-024), Knock-Out Serum Replacement (KSR; Cat# 10828-028), 10 mM non-essential amino acids (NEAA; Cat# 11140-050), sodium pyruvate (Cat# 11360-070), penicillin–streptomycin (Pen/Strep; Cat# 15140-122), and cell culture-grade water (Cat# 15230-162). TrypLE™ Express (Cat# 12605-028) was used for cell dissociation. Retinal differentiation (RD) was induced with CHIR99021 (GSK3β inhibitor; Stemgent, Cat# 04-0004-02), and PD0325901 (MEK/ERK inhibitor; Stemgent, Cat# 04-0006-02). For optic vesicle and photoreceptor development, we used TOM’s medium supplemented with N2 (Gibco™, Cat# 17502-048), B27 (Gibco™, Cat# 17504044), all-trans retinoic acid (Sigma-Aldrich, Cat# R2625-50MG), taurine (Sigma-Aldrich, Cat# T-8691), and GFR Matrigel® (Corning®, Cat# 356,230) (Supplementary Table S1). Cultureware included 96-well ultra-low-adhesion Lipidure-Coat plates (AMSbio, Cat# AMS.51011610), 6-well ultra-low-adhesion plates (Corning™, Cat# 07-200-601), and sterile glass Pasteur pipettes. For metabolite extraction and derivatization, methanol, chloroform, water and pyridine (all ≥ 99%, Sigma-Aldrich), methoxyamine hydrochloride (Thermo Fisher), and BSTFA (CS-Chromatographie Service GmbH, Langerwehe, Germany, ≥ 98.5%) were used.

### Study design and workflow

Neonatal C57BL/6 retinas (postnatal day 3) and day 28 MROs differentiated from ESD3 mouse embryonic stem cells (ESCs) were processed in parallel, derivatized and analyzed by gas chromatography electron ionization quadrupole mass spectrometry (GC–EI–Q–MS).

**Fig. 1:**
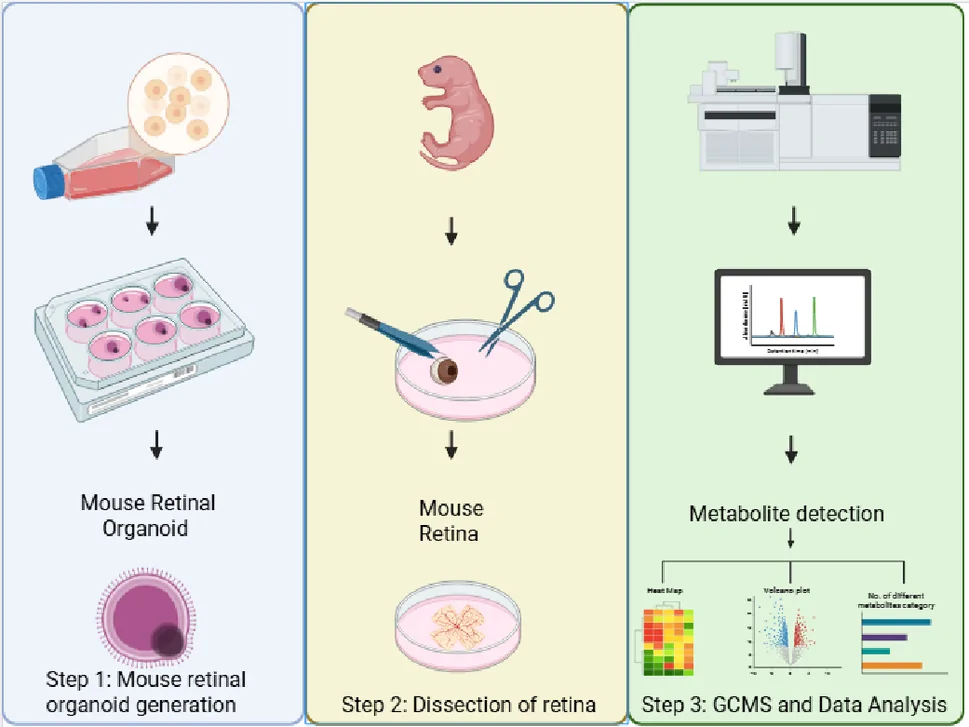
Schematic overview of the workflow for generating and metabolic analysis of MROs. The left panel (Step 1) shows the generation of MROs from ESD3 line under controlled conditions to fully differentiated MROs. Middle panel (Step 2) shows the isolation of native retina from neonatal wild type mice via eyeball excision and dissection. The right panel (Step 3) shows GCMS setup followed by metabolomic analysis. (Created with BioRender. Gupta (2026) https://BioRender.com/gwz46yw)

We performed an untargeted, comparative metabolomic analysis to assess how closely stem cell-derived MROs recapitulate the endogenous metabolome of mouse retina in three steps (Fig. 1). Three independent biological replicates were analyzed for both mouse retina and retinal organoids. Retinal organoids originated from three independent differentiation batches to minimize the impact of batch-specific variability.

### Sample preparation

#### Animals

All animal experiments were conducted according to German animal welfare law and approved by the authorities of the State of Saxony. Experiments were performed using retinas from P3 female and male C57BL/6 (Jackson Lab). Eyes were enucleated and the retinas were subsequently dissected under a sterile stereomicroscope and cultured in DMEM (Gibco™, Cat# 10564-011).

#### MROs differentiation

Mouse ESD3 embryonic stem cells (ESCs) were seeded at a density of 3,000 cells per well in ultra-low-adhesion U-bottom 96-well plates using RD medium. On day 1, 2% (v/v) Matrigel was added to each well to provide laminin- and collagen-rich basement membrane signals essential for optic vesicle morphogenesis and the establishment of epithelial polarity. During the induction phase (days 0–7), organoids were maintained in RD medium to promote early neuroectodermal differentiation and optic vesicle-like structure formation. From days 7–15 (patterning phase), organoids were transferred to 6-well suspension culture plates and maintained in TOM’s medium supplemented with N2 and B27, supporting retinal progenitor survival and photoreceptor lineage commitment. Between days 10–15, the medium was additionally supplemented with 0.5 µM retinoic acid and 1 mM taurine to enhance photoreceptor specification and protect against oxidative stress. From days 15–28 (maturation phase), organoids were cultured in plain TOM’s medium without any supplements to promote terminal differentiation and laminar organization of retinal tissue (Supplementary Table S1). Conditioned media samples were collected longitudinally from the independent retinal organoid differentiation batches at defined developmental stages (day 8–28). Each developmental time point represents metabolite profiles obtained from the corresponding organoid culture batch.

### Quantitative real-time PCR (qPCR) analysis

#### RNA isolation and cDNA synthesis

Total RNA was isolated from MROs using the NucleoSpin RNA Mini Kit (Macherey–Nagel, #740955.250) according to the manufacturer’s protocol with minor modifications. Briefly, five MROs pooled from cuture per samples (n=3) were collected in 1.5 mL microcentrifuge tubes, washed once with 1× phosphate-buffered saline (PBS), and residual buffer was aspirated. Samples not processed immediately were snap-frozen and stored at − 80 °C until further use. For RNA extraction, organoids were lysed in RA1 buffer and mechanically homogenized by pipetting (10 repetitions). On-column rDNase digestion was performed to eliminate genomic DNA contamination. RNA was eluted in 30 µL RNase-free water to account for low input material. RNA concentration and purity (A260/280 and A260/230 ratios) were assessed using a NanoDrop spectrophotometer (DeNovix), with RNase-free water as blank. Complementary DNA (cDNA) was synthesized using the iScript™ cDNA Synthesis Kit (Bio-Rad, #170-8891). RNA was diluted in nuclease-free water to a final volume of 15 µL. Reverse transcription reactions were prepared according to the manufacturer’s instructions to a final volume of 20 µL. The reaction conditions were as follows: priming at 25 °C for 5 min, reverse transcription at 46 °C for 20 min, and enzyme inactivation at 95 °C for 1 min. The resulting cDNA was diluted 1:6 in nuclease-free water and stored at − 20 °C until further use.

#### Analysis

Quantitative real-time PCR (qPCR) was performed using GoTaq® qPCR Master Mix (2×; Promega, #A600A). The reaction mixture consisted of PCR-grade water, 10 µM forward and reverse primers, and 1× master mix. Technical triplicates were performed. Gene expression analysis was conducted for the following targets: *Pax6, Prkca,* and *Opn1sw*. Primer sequences are provided in Supplementary Table S2. For each reaction, 5 µL of cDNA template was combined with 7.5 µL of qPCR master mix in a 384-well plate, which was subsequently sealed with adhesive PCR film. Amplification was performed using the QuantStudio™ 5 Real-Time PCR System (Applied Biosystems, Thermo Fisher Scientific) under standard cycling conditions (Supplementary Table S3). Quantification cycle (Ct) values were obtained using QuantStudio Design & Analysis Software v1.4.3. Relative gene expression was calculated using the comparative ΔΔCt method, with *ACTB* serving as the endogenous reference gene. Expression levels were normalized to undifferentiated mouse embryonic stem cells (mESCs).

### Immunofluorescence staining and microscopy

Protein expression was evaluated by immunofluorescence using MROs collected at day 28 of differentiation. MROs were fixed in 2% paraformaldehyde, cryoprotected in sucrose, embedded in OCT compound, and cryosectioned at a thickness of 12 μm. Sections were washed in cold phosphate-buffered saline (PBS) and permeabilized and quenched using a glycine solution containing 0.1% Triton X-100. Non-specific antibody binding was blocked for 1 h at room temperature using blocking buffer containing 1% bovine serum albumin (BSA), 1% normal donkey serum, 0.1% Triton X-100, and 0.1% Tween-20 in PBS. Primary antibodies were diluted in blocking buffer and incubated overnight at 4 °C. Following permeabilization and blocking, organoid sections were incubated with primary antibodies (24 h) against ARR3 (goat, Ab-A55, 1:500), CRX (sheep, SCTC, 1:50), NRL (goat, Ab-N40, 1:500), CALBINDIN (rabbit, Abcam, ab108404, clone EP3478, 1:150), PROX1 (rabbit, Abcam, ab199359, clone EPR19273, recombinant, 1:500), and glutamine synthetase (GS) (rabbit, Abcam, ab176562, clone EPR13022(B), 1:250). Additional antibodies used for retinal characterization included, and recoverin (rabbit, Ab-R99, 1:1000). Following PBS washes, sections were incubated with species-specific Alexa Fluor-conjugated secondary antibodies for 1 h at room temperature in the dark including donkey anti-goat Alexa Fluor 488 (1:500), donkey anti-sheep Alexa Fluor 568 (1:500), , ( donkey anti-rabbit Alexa Fluor 488 (1:500), and Donkey anti-Goat Alexa Fluor 680 (1:500) .Cell nuclei were counterstained with DAPI (1:100), and sections were mounted using fluorescence mounting medium. Fluorescence images were acquired using a fluorescence microscope (ZEISS Axio Imager Z1 Fluorescence Microscope) equipped with appropriate filter sets for DAPI and Alexa Fluor fluorophores. Approximately 4–5 cryosections were examined for each experimental condition, and representative images are presented in the Results section.

### Metabolite extraction and derivatization

#### Extraction

We developed a pipeline for extraction of metabolites based on a previously established protocol (Du et al., 2016). For intracellular metabolomic analysis, one biological replicate consisted of a single retinal organoid generated from an independent differentiation batch or a single retina isolated from one P3 mouse (male or female). Retinas were processed individually. Three independent biological replicates (n = 3) were analyzed for each group. To standardize biological input, one retinal organoid and one native retina were processed as individual biological samples for comparative metabolomic analysis. Each biological replicate was homogenized in 140 µL of ice-cold methanol:chloroform:water (70:20:5, *v/v*) buffer by gentle pipetting for 2–5 min, vortexed to enhance metabolite solubilization. After incubation on ice for 15 min, the samples were centrifuged at 14,000×*g* for 15 min at 4 °C. The complete supernatant was collected and lyophilized.

#### Derivatization

Dried extracts were first methoxyaminated by adding 25 µL of 20 mg/mL methoxyamine hydrochloride in pyridine and incubated at 37 °C for 90 min. Subsequently, 46 µL of *N,O*-bis(trimethylsilyl)trifluoroacetamide (BSTFA) was added for silylation at 70 °C for 30 min. The derivatized samples were transferred to 1.5 mL GC vials fitted with 0.2 mL glass microinserts and sealed.

### GC–EI–Q–MS analysis

Samples were analyzed using an Agilent 6890/5973N Plus GCMS system, coupled with electron impact (EI) ionization and a quadrupole mass spectrometer (HP-MSD). This setup enabled high-sensitivity detection and identification of semi-volatile metabolites. One microlitre of each sample was injected in splitless mode at 280 °C. Metabolite separation was achieved on a DB-5MS Ultra Inert column (30 m × 0.25 mm ID, 0.25 µm film; Agilent Technologies, Germany) using helium 5.0 (Air Liquide, Germany) as the carrier gas at 1.2 mL/min. The oven program started at 40 °C (1 min hold), ramped to 325 °C at 6 °C/min, and was held for 9 min. Electron impact ionization was performed at 70 eV with the ion source set to 250 °C. Mass spectra were acquired over *m/z* 70–800. The instrument was quality-tested with a reference mix before running the experiment. We used a fresh tune to establish standard conditions and analysis of blanks to ensure suitable analytical conditions, in particular potential contamination and sensitivity of the instrument. Initial experiments were carried out to, inter alia, explore the expected general appearance of the chromatograms which was observed throughout all sample runs. Samples were randomized and blanks were run throughout the sequence to minimize the risk of cross contamination.

### Data processing

Raw chromatograms were processed using AMDIS 2.71 for deconvolution (Stein, 1999). Peaks were annotated by manually curated, automated mass spectral comparison with the NIST14 mass spectral library (National Institute of Standards and Technology, Massachusetts, USA), with retention indices computed using chromatogram-intrinsic retention time standards. Only metabolites with high-confidence spectral matches (similarity score ≥ 800) were retained. Quantitative integration of manually selected ion fragments (*m/z* ± 0.5) was performed using Mass Hunter Quantitative Analysis B.07.00 (Supplementary Excel Sheet 1). Chromatographic quality was assessed by inspection of baseline stability, peak shape, and retention-time alignment across all samples. Total ion chromatograms showed consistent retention times and comparable signal quality throughout the analytical sequence, supporting stable instrument performance and consistent sample processing.

### Statistical analysis

Peak area data obtained from GCMS analysis were imported into the MetaboAnalyst 6.0 (www.metaboanalyst.ca/*)* (accessed May 2026) for statistical evaluation. Data preprocessing included median normalization, missing value imputation using the minimum detected value, log10 transformation, and auto-scaling. Metabolites with FDR < 0.05 and a fold change (FC) ≥ 10, or ≤ 0.1 were considered significantly altered. Fold change analysis is provided in Supplementary Excel Sheet 2. Differential metabolite abundance between mouse retina and MROs was assessed using unpaired Student’s t-test with Benjamini–Hochberg FDR correction (Supplementary Excel Sheet 3) and one-way ANOVA with post hoc analysis (Fisher’s LSD) for metabolomic analysis of conditioned media collected at different developmental stages of MROs(Supplementary Excel Sheet 4). Statistical analyses for qPCR were performed with the GraphPad Prism 10.4.1 software. Microscopy image acquisition was performed using a ZEISS Axio Imager fluorescence microscope, and the images were analysed using ZEN 3.8 software (ZEISS). The DAPI channel was set to be the reference focus. Heatmaps based on significantly different metabolites between retinas and organoids were generated using hierarchical clustering of averaged replicates without autoscaling, using Euclidean distance and Ward’s linkage.

#### Pathway analysis

Metabolites identified by mass spectral matching against the NIST library were assigned KEGG compound identifiers (Kyoto Encyclopedia of Genes and Genomes (KEGG)) and, submitted to MetaboAnalyst 6.0 for pathway topology analysis (www.metaboanalyst.ca; accessed May 2026).

## Results

### Characterization and chromatogram of mouse retinal organoid

**Fig. 2:**
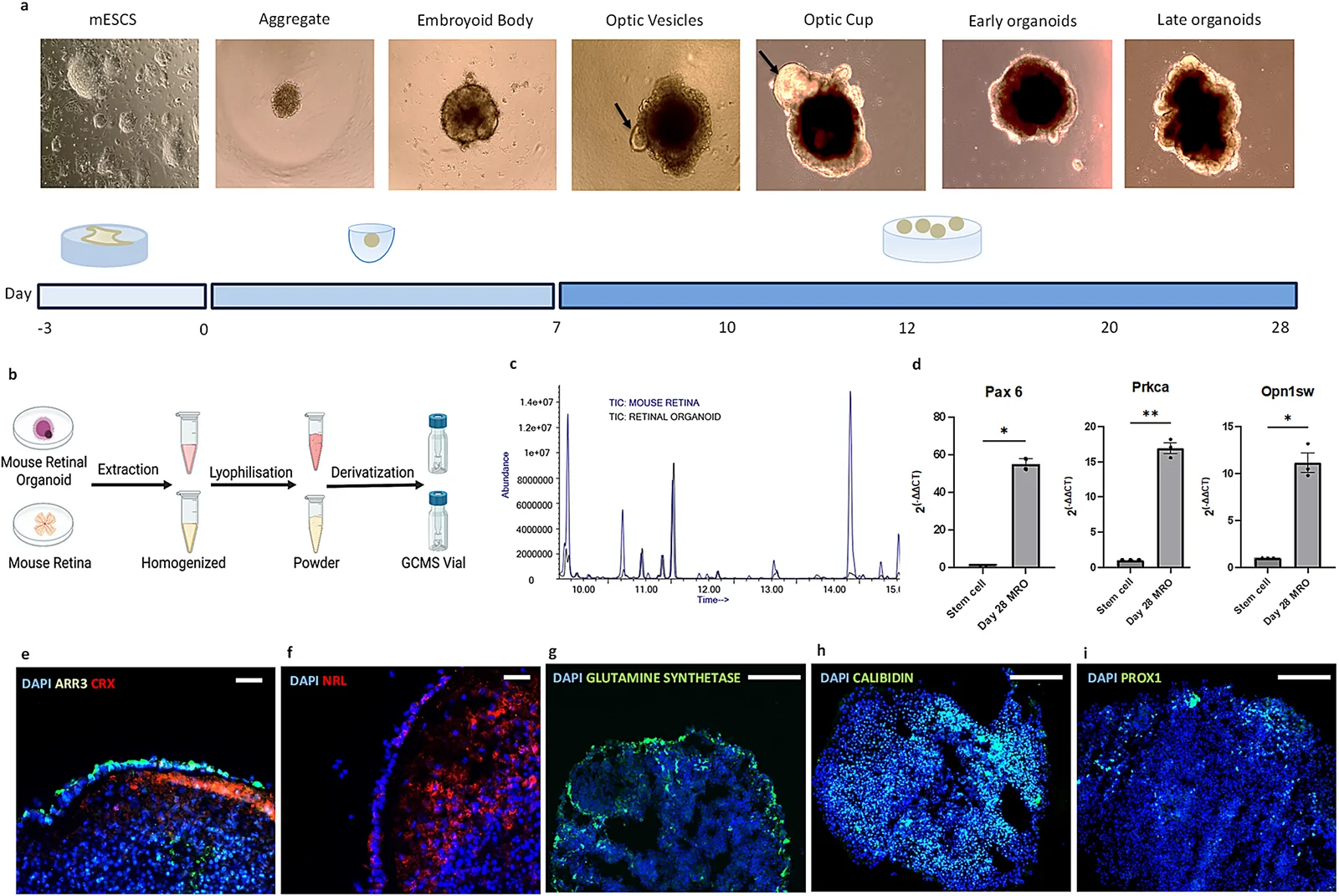
MROs generation, processing, total-ion GCMS chromatograms and cell type characterization. **a** Representative bright-field images illustrating key stages of retinal organoid differentiation from mouse embryonic stem cells (mESCs) to late organoids (day 28) (Bio Protocols) Anna La Torre, 2016 and **b** schematic of MROs and mouse retina tissue processing for metabolomic analysis (Created in BioRender. Gupta (2026) https://BioRender.com/nc3z5cj). **c** Overlay of total-ion chromatograms (TICs) of mouse retina (blue trace) and MROs (black trace). **d** qPCR analysis showing relative expression of Pax6, Prkca, and Opn1sw in day-28 MROs. Data are presented as mean ± SEM from three independent biological replicates. Statistical significance was determined using unpaired t-tests and is indicated as follows: ns, not significant (P > 0.05); *P ≤ 0.05; **P ≤ 0.01. **e**–**i** Immunofluorescence characterization of day-28 mouse retinal organoids. Representative images showing expression of photoreceptor-associated markers: ARR3 (green), CRX (red), and NRL (red); Müller glial marker glutamine synthetase (green); and retinal interneuron-associated markers: CALBINDIN (green) and PROX1(green). Nuclei were counterstained with DAPI. Scale bars: 50 μm for the ARR3/CRX and NRL panels; 100 μm for the glutamine synthetase, CALBINDIN, and PROX1 panels

MROs were generated from mouse embryonic stem cells (mESCs) using a stepwise differentiation protocol recapitulating major stages of retinal development, including embryoid body formation, optic vesicle emergence, optic cup morphogenesis, and retinal maturation (Fig. 2a). GC–MS metabolomic profiling was performed using three independent biological replicates per group (n = 3), each consisting of one retinal organoid derived from an independent differentiation batch or one individually processed P3 mouse retina. Equal biological input (one retinal organoid versus one retina per replicate) was used throughout the study. Afterwards all the samples underwent extraction and derivatization (Fig. 2b). The selection of day-28 MROs and P3 mouse retina was guided by previous transcriptomic studies showing that MROs at this stage most closely resemble early postnatal retinal development rather than the mature adult retina (Cui et al., 2020). Total ion chromatograms demonstrated broadly similar metabolic profiles between MROs and mouse retina, with aligned retention times across major peaks, indicating preservation of core retinal metabolic features (Fig. 2c). Differences in peak intensities were observed for multiple metabolites between two sample types. Supportive qPCR characterization confirmed retinal lineage differentiation in day 28 organoids (Fig. 2d). PAX6 (retinal progenitor cell marker), OPN1SW (cone photoreceptor marker), and Prkca (bipolar cell marker) expression levels were significantly increased compared with undifferentiated mESCs. Immunofluorescence analysis further supported retinal differentiation of day-28 mouse retinal organoids (Fig. 2e-i). ARR3 (cone photoreceptors), CRX (photoreceptor lineage), NRL (rod photoreceptor) immunoreactivity was predominantly observed in the outer region of the MROs, while immunoreactivity for glutamine synthetase (Müller glial cells), CALBINDIN (horizontal cells), and PROX1 (retinal interneurons) was also detected, demonstrating expression of markers associated with multiple retinal cell types.

### Classification of metabolites tentatively identified in mouse retina and retinal organoids

**Fig. 3:**
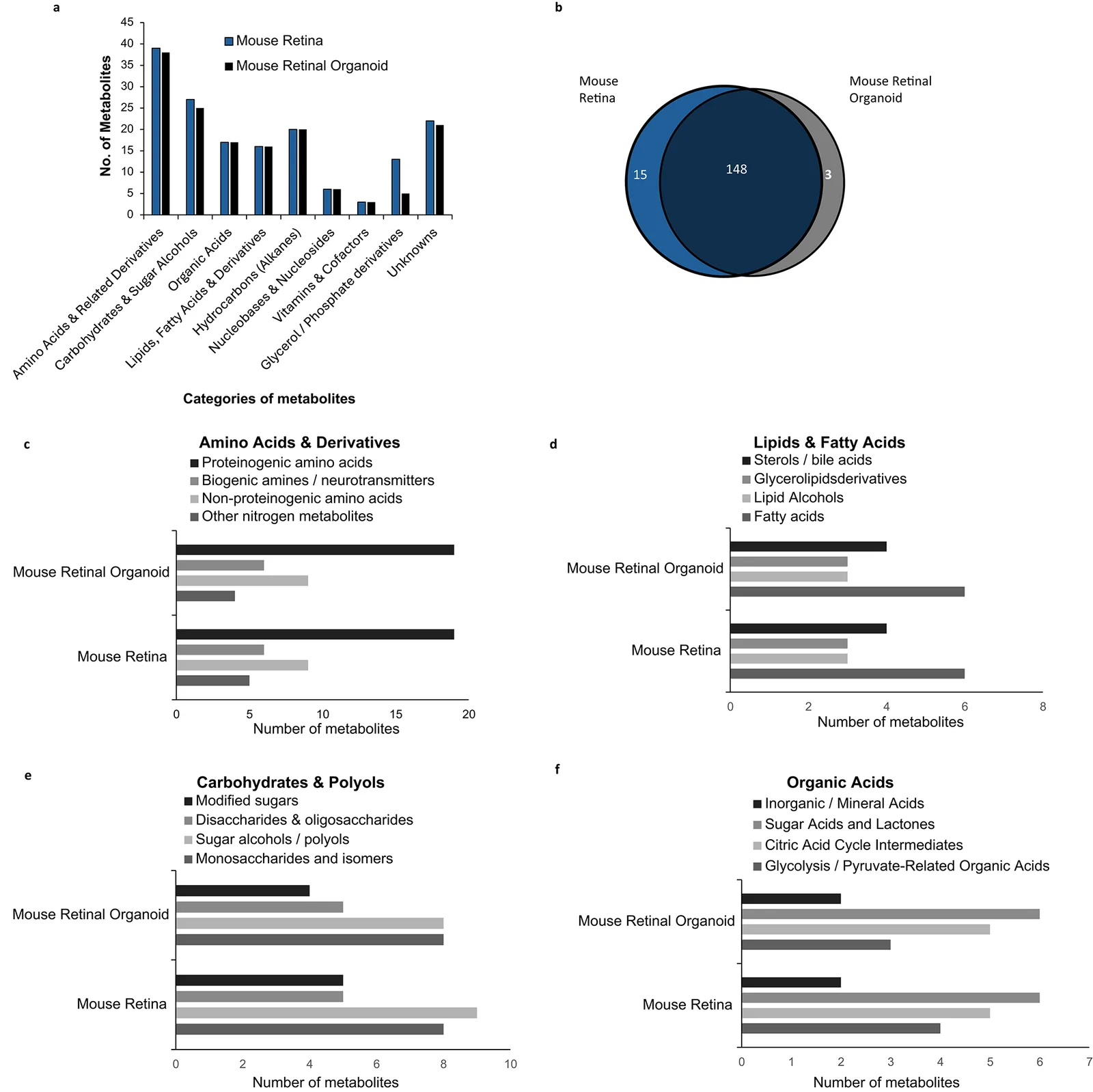
Comparative classification of detected metabolites in mouse retina and MROs. **a** Distribution of annotated metabolites across major biochemical categories in mouse retina (blue) and retinal organoids (black). **b** Venn diagram showing shared and unique metabolites between mouse retina and MRO. **c**–**f** Subclassification of metabolites within amino acids and related derivatives, lipids and fatty acids, carbohydrates and polyols, and organic acids in mouse retina and MRO. Bars represent the number of metabolites detected in each category

Detected compounds from mouse retina and MROs were classified into major biochemical categories (Fig. 3a). A total of 148 annotated metabolites was common to both systems, whereas 15 metabolites were detected in mouse retina but were below the detection limit in MROs and 3 metabolites were detected in MRO but were below the detection limit in mouse retina (unknown 275, unknown 400, and unknown 354) only in MROs (Fig. 3b). The annotated metabolites were grouped into amino acids and related derivatives, lipids and fatty acid derivatives, carbohydrates and sugar alcohols, and organic acids (Fig. 3c–f).

Amino acid-related metabolites represented the largest metabolite class in both systems, followed by carbohydrates/sugar alcohols and lipid-associated metabolites. Organic acids, including glycolysis- and TCA-cycle-associated metabolites, were also detected in both mouse retina and MRO. Overall, the distribution of the metabolite classes demonstrated a broad metabolic overlap between MRO and native mouse retina.

### Unsupervised and supervised clustering of metabolites in MRO

**Fig. 4:**
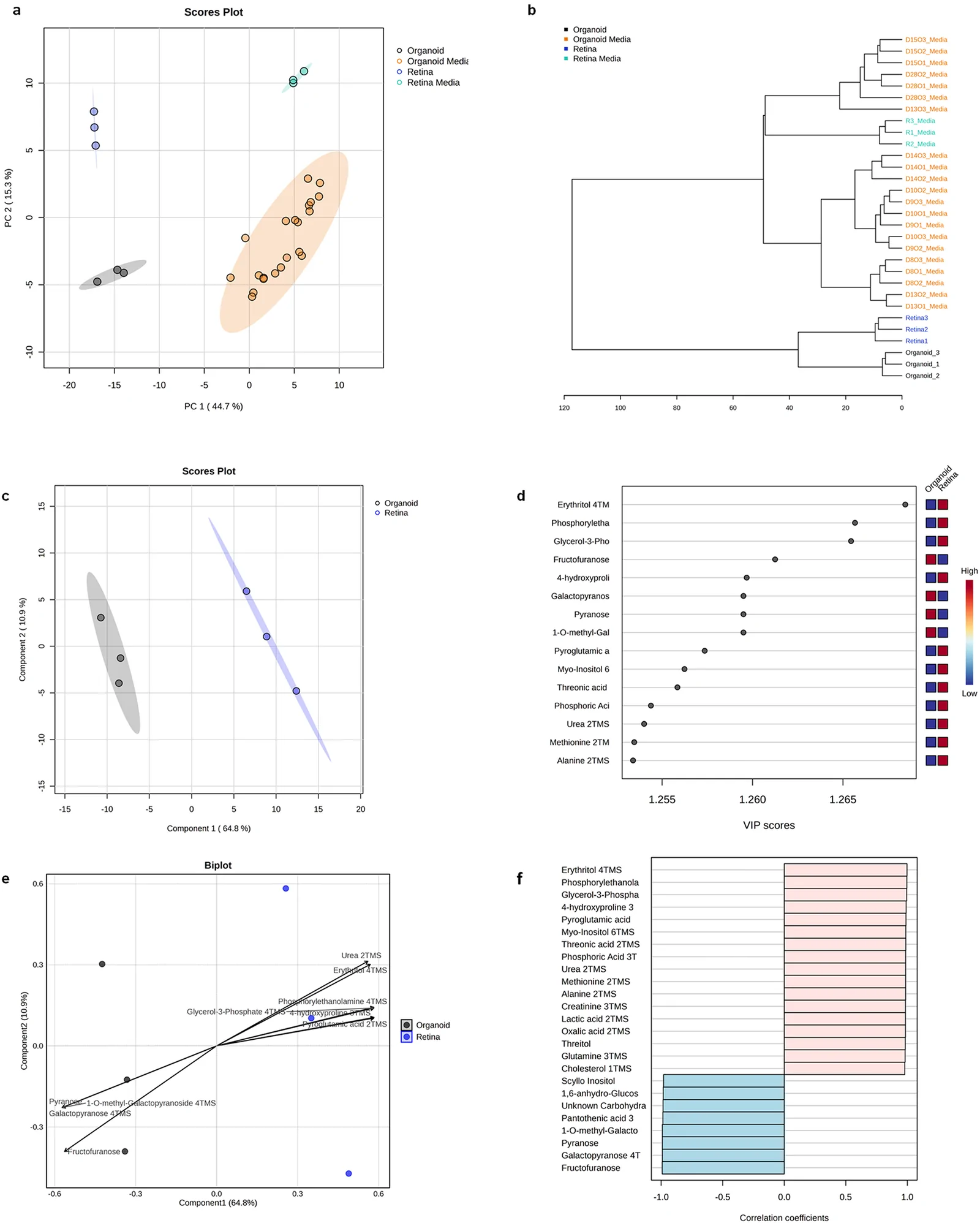
Clustering metabolites in mouse retina and MRO. **a** PCA score plot generated from normalized GCMS metabolite intensities of mouse retina, MRO, mouse retina-conditioned medium, and MRO-conditioned media collected across different developmental stages. **b** Hierarchical clustering dendrogram constructed using distance-based clustering of metabolite profiles of mouse retina, MRO, and their culture media. **c** PLS-DA score plot showing separation between MRO and mouse retina. **d** VIP scores identifying metabolites that most strongly drive group separation between retinal organoids and retina. **e** Biplot showing metabolite distribution between MRO and native retina samples. Arrows represent metabolite loading vectors indicating metabolites contributing to sample separation. **f** Pattern correlation the top positive (pink) and negative (blue) correlated metabolites associated with the MRO–retina comparison

Unsupervised multivariate analyses were performed to compare the metabolic profiles of mouse retina, day-28 MRO, mouse retina-conditioned medium and MRO-conditioned media collected across early (day 8-10), intermediate (days 13–15), and late (day 28) developmental stages, with three biological replicates per group (n = 3). Principal component analysis (PCA) demonstrated clear separation between tissue and media samples, with PC1 accounting for 44.7% of the total variance and PC2 explaining 15.3% (Fig. 4a).

Mouse retina and MROs formed distinct clusters, while the corresponding media samples clustered separately. Hierarchical clustering analysis further confirmed group-specific segregation , with biological replicates clustering closely within each group (Fig. 4b). Partial least squares–discriminant analysis (PLS-DA), a supervised multivariate analysis, further demonstrated distinct separation between mouse retina and MRO samples (Fig. 4c). According to variable importance in projection (VIP) analysis, several metabolites contributed strongly to group discrimination (VIP > 1), including pantothenic acid, erythritol, phosphate, glycerol-3-phosphate, and carbohydrate-associated metabolites (Fig. 4d). The PLS-DA biplot of metabolomic profiles from MRO and mouse retina indicated the strong group separation. Metabolites with vectors directed toward mouse retina cluster were urea, erythritol, phosphoethanolamine, and pyroglutamic acid, whereas metabolites directed toward the MRO cluster were fructofuranose, galactopyranose, and 1-O-methyl-galactopyranoside (Fig. 4e). Pattern correlation analysis identified coordinated metabolite relationships between mouse retina and MRO (Fig. 4f). Among the 148 annotated metabolites, 28 showed significant correlations at a stringent threshold of FDR-adjusted < 0.005, including 19 positively correlated and 9 negatively correlated metabolites.

### Differential metabolite abundance patterns of MRO

**Fig. 5:**
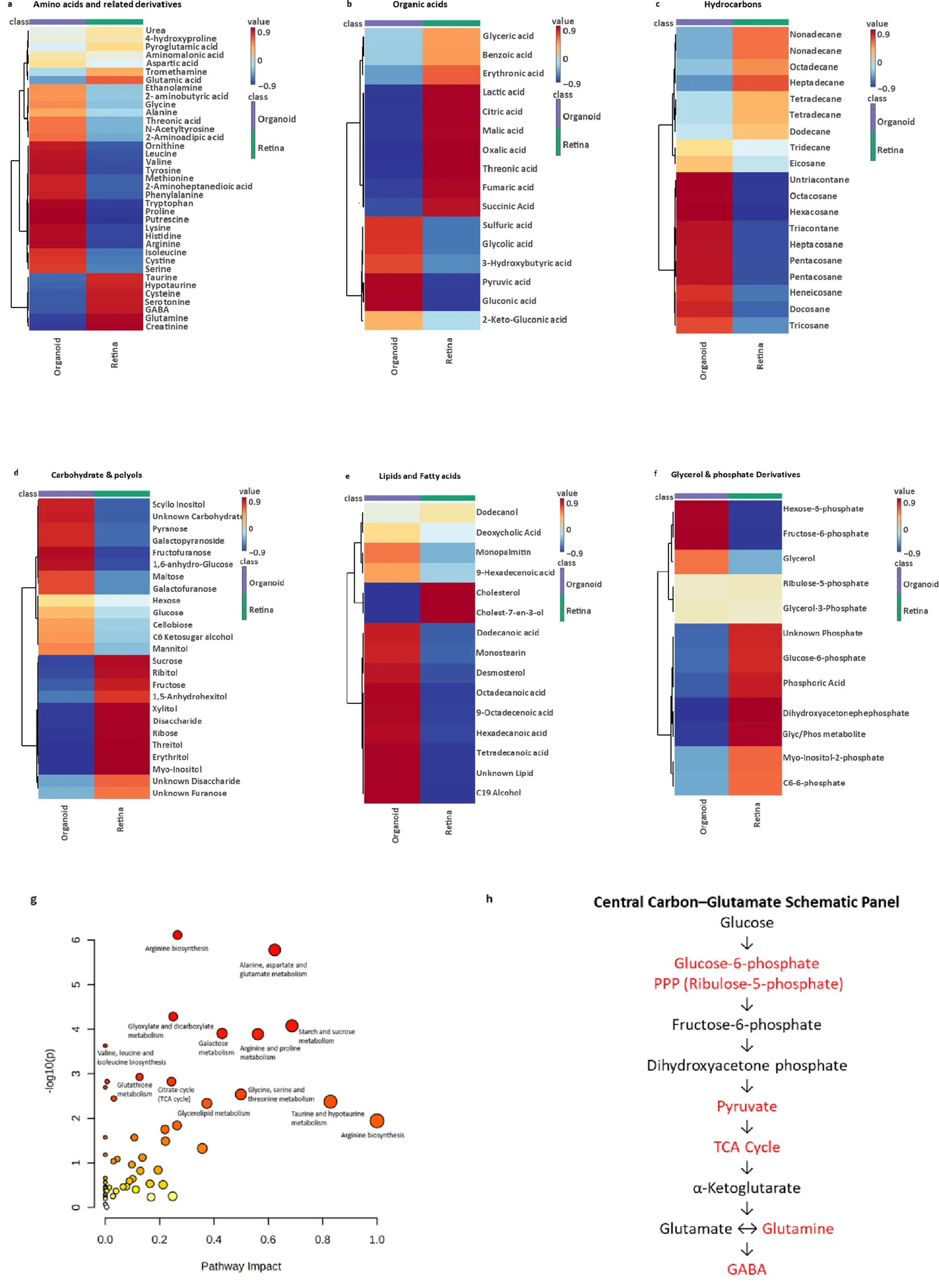
Hierarchical clustering heatmaps and pathway analysis in mouse retina and MROs. Clustered heatmaps of **a** amino acids and related derivatives, **b** organic acids, **c** hydrocarbons, **d** carbohydrates and polyols, **e** lipids and fatty acids, and **f** glycerol/phosphate derivatives. Rows represent metabolites and columns represent mean values across biological replicates. Heatmaps display relative abundances with unsupervised clustering applied to both metabolites and samples. **g** Pathway analysis showing altered abundance of metabolites associated with pathways based on pathway impact and statistical significance (–log10 p-value). **h** Schematic proposing metabolites associated with carbon metabolism, contributing to neurotransmitter in MROs. Red color indicates metabolites with reduced abundance.

Heatmap analysis demonstrated distinct metabolic clustering between mouse retina and MRO across amino acids, organic acids, hydrocarbons, carbohydrates, lipids, and phosphate-associated metabolites (Figs. 5a–f, S3 and Supplementary Excel Sheet 3 ). Amino acid and neurotransmitter-associated metabolites, including glutamine, taurine, hypotaurine, and glutamate-related metabolites, generally showed higher abundance in mouse retina, whereas selected amino acid derivatives were relatively enriched in MRO. Organic acid profiling revealed differential abundance of glycolysis and TCA-cycle-associated metabolites, with several glycolytic intermediates reduced in MRO. Carbohydrate profiling demonstrated elevated abundance of several monosaccharides and modified sugars in MRO, while phosphorylated carbohydrate intermediates, including glucose-6-phosphate and related phosphate-containing metabolites, showed lower abundance compared to mouse retina. Lipid and sterol profiling also revealed distinct group-specific abundance patterns between mouse retina and MRO.

Pathway analysis identified significant alterations in arginine biosynthesis, starch, and sucrose metabolism, as well as glycine, serine, and threonine metabolism, and taurine/hypotaurine metabolism (Fig. 5g). We propose an altered model of metabolite abundance for MROs based on the metabolomic differences identified in this study: compared with native mouse retina, MROs exhibited reduced levels of metabolites associated with glycolysis, the pentose phosphate pathway, the TCA, and glutamine/GABA metabolism (Fig. 5h).

### Metabolite profiles in conditioned media during MRO development

**Fig. 6:**
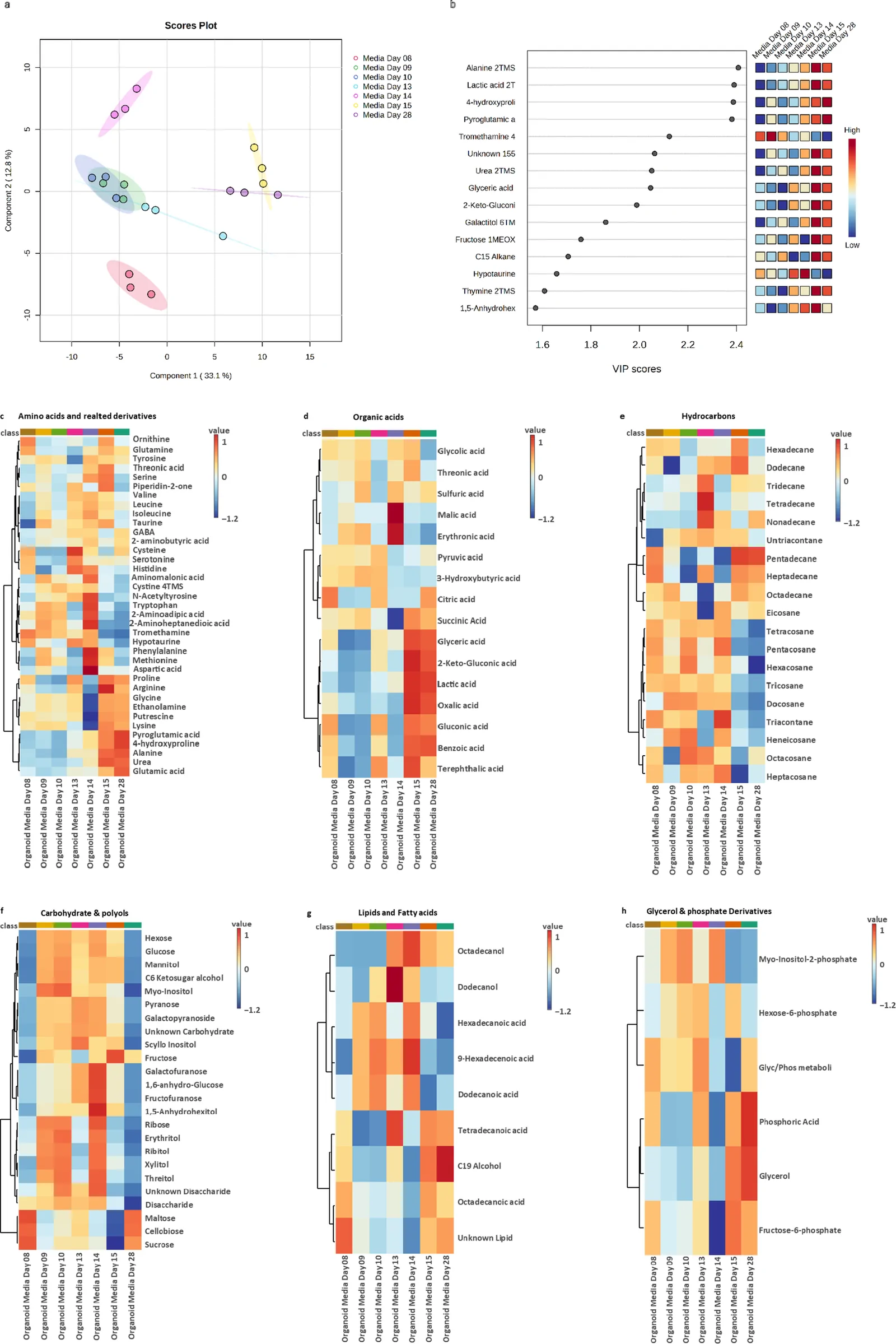
Multivariate analysis and hierarchical clustering of the MRO-conditioned media at different developmental stages. **a** PLS-DA score plot showing the separation MRO at different developmental stages. **b** Variable Importance in Projection (VIP) scores identifying metabolites that most strongly drive group separation between MRO conditioned media at different developmental stages. Hierarchical clustering heatmaps of major metabolite subclasses in MROs conditioned media at different days of collection: **c** Amino acids and related derivatives, **d** organic acid, **e** hydrocarbons, **f** carbohydrates and polyols, **g** lipids and fatty acids, and **h** glycerol/phosphate derivatives. Rows represent metabolites and columns represent mean metabolite abundances of three biological replicates (n=3) at each developmental time point.

GCMS metabolomic profiling of conditioned media collected during retinal organoid maturation (day 8 to day 28) revealed temporal changes in extracellular metabolite composition (Figs. 6, S2 and Supplementary Excel Sheet 4 ). Multivariate analysis demonstrated clear stage-dependent clustering of media samples, with early (day 8–10), intermediate (day 13–15), and late (day 28) stages forming distinct groups (Fig. 6a). PLS-DA VIP analysis identified several metabolites contributing strongly to stage separation (Fig. 6b). Hierarchical clustering heatmaps revealed stage-specific remodeling across amino acid, organic acid, carbohydrate, lipid, hydrocarbon, and phosphate-associated metabolites during organoid maturation (Fig. 6c–h). Hierarchal clustering heatmaps further demonstrated temporal changes in metabolite abundance, with several amino acid–related metabolites showing increased abundance at later developmental stages. Organic acid profiles demonstrated pronounced temporal clustering, while metabolites associated with central carbon and carbohydrate metabolism displayed differences in abundance during later stages. Lipids and hydrocarbons exhibited comparatively moderate variation across developmental stages. Glycerol and phosphate-associated metabolites showed distinct fluctuations and clustering patterns across maturation time points.

## Discussion

This study aimed to characterize the biochemical profile of MROs to evaluate their potential as an alternative experimental model to mouse retina. Organoid generation is faster and more efficient in terms of material consumption, and it supports the 3R principles (replacement, reduction, and refinement) by minimizing the use of living animals in research. 

 We discovered that MROs exhibited a substantial retinal metabolic signature despite selective metabolic divergence compared to mouse retina. A broad overlap was observed across amino acids, neurotransmitter-associated metabolites, glycolytic intermediates, TCA-cycle metabolites, lipids, sterols, and nucleotide-associated compounds, indicating that MROs recapitulate major components of retinal biochemical organization.

### Conserved retinal metabolic core and selective metabolic divergence

A major finding of this study is that MROs recapitulate a substantial proportion of annotated metabolites with the native mouse retina as detected by GCMS . Conserved metabolites included neurotransmitter-associated compounds (glutamate, gamma-aminobutyric acid (GABA), and taurine), amino acids (glutamine, glycine, alanine, serine, methionine, valine, leucine, and phenylalanine), and TCA-cycle intermediates (citrate, succinate, fumarate, and malate), indicating substantial overlap in metabolites associated with retinal neuronal function and energy metabolism. Taurine, one of the most abundant metabolites in the vertebrate retina, is essential for retinal development and photoreceptor maintenance (Ripps & Shen, 2012), whereas glutamate and GABA are central mediators of retinal neurotransmission (Du et al., 2016). Together, these findings suggest that MROs develop a retina-like biochemical identity rather than representing metabolically unrelated in vitro tissue systems.

Despite this broad similarity, several metabolites associated with glycolysis, pentose phosphate metabolism, phospholipid metabolism, and phosphate-associated pathways were detected in native retina but were below the detection limit in MROs, including glucose-6-phosphate, glycerol-3-phosphate, dihydroxyacetone phosphate, ribulose-5-phosphate, phosphoethanolamine, and myo-inositol. These observations suggest divergence in abundance of central carbon metabolism and membrane-associated metabolic pathways. However, because GC–MS detection is influenced by metabolite concentration, extraction efficiency, and derivatization yield, metabolites not detected in organoids should not be interpreted as completely absent (Fiehn, 2016).

### Central carbon metabolism, neurotransmitters, and lipid metabolism

GC–MS metabolomic profiling revealed that mouse retinal organoids recapitulate a substantial proportion of the annotated metabolic landscape of the native retina while exhibiting selective differences in metabolites associated with energy metabolism, phospholipid metabolism, and neurotransmitter biosynthesis. Multivariate analyses demonstrated a clear separation between organoids and retina; however, most most annotated metabolites were shared between the two tissues, indicating a similarity of a core retinal metabolic program. Retinal metabolism is highly dependent on coordinated glycolytic and mitochondrial pathways to support photoreceptor function, neurotransmitter recycling, and cellular homeostasis (Aït-Ali et al., 2015; Chinchore et al., 2017; Hurley et al., 2015). The altered abundance of glucose-associated metabolites, phosphorylated sugar intermediates, pyruvate-related metabolites, and TCA-cycle-associated metabolites observed in MROs is therefore consistent with differences in central carbon metabolism relative to native retina. Likewise, reduced abundance of glutamate, aspartate, and arginine may reflect differences in neurotransmitter-associated metabolism and neuron–glia metabolic coupling, which are essential features of mature retinal tissue (Bringmann et al., 2013). Differences were also observed in cholesterol, desmosterol, fatty acids, and phospholipid-associated metabolites. Lipid metabolism plays a critical role in membrane biosynthesis, photoreceptor outer segment formation, and synaptic organization (Fliesler & Bretillon, 2010). Pathway enrichment analysis further supported coordinated altered abundance of alanine–aspartate–glutamate metabolism, arginine biosynthesis, arginine and proline metabolism, glyoxylate and dicarboxylate metabolism, and starch and sucrose metabolism. Collectively, these pathways highlight selective differences in abundance of amino acid-associated and carbohydrate-associated metabolism between organoids and native retina (Xia & Zhang, 2022). The altered abundance of lipid- and sterol-associated metabolites may therefore be suggestive of differences in the metabolic maturation of photoreceptors under current culture conditions. Nevertheless, conservation of several fatty acid-associated metabolites indicates that components of lipid metabolism are represented in MROs and mouse retina (Fliesler & Bretillon, 2010).

### Temporal changes in the conditioned media during retinal organoid maturation

Analysis of conditioned media demonstrated temporal changes in extracellular metabolic profiles during retinal organoid maturation. Temporal alterations were particularly evident in amino acid-associated and carbohydrate-associated metabolites between early and late developmental stages. Previous studies have shown that developing retinal tissue and retinal organoids actively consume glutamate and aspartate during maturation (Li et al., 2020), consistent with the patterns observed here. These findings support the concept of a retinal metabolic ecosystem in which metabolite exchange contributes to tissue development and homeostasis (Hanna et al., 2025; Kanow et al., 2017).

### Implications for organoid optimization and future directions

The substantial overlap between MROs and native retina supports the utility of organoids as experimentally accessible models for investigating retinal development, inherited retinal degeneration, metabolic retinal disorders, and therapeutic interventions. At the same time, the metabolic differences identified in this study highlight potential candidates for improving organoid maturation. Optimization of nutrient composition, oxygen availability, long-term culture conditions, retinal pigment epithelium interaction, and co-culture systems may improve metabolic fidelity and photoreceptor maturation. Future integration of metabolomics with transcriptomics, isotope-tracing approaches, and functional assays will further advance the understanding of retinal organoid metabolic development. Recent studies increasingly emphasize that successful retinal organoid maturation requires not only appropriate cellular differentiation but also establishment of retina-specific metabolic programs and intercellular metabolic interactions (Fu et al., 2021; Hanna et al., 2025; Joyal et al., 2018). The present findings contribute to this growing body of evidence by identifying both conserved retinal metabolic features and areas of selective metabolic divergence between MROS and Mouse retina.

### Limitations of the study

Retinal organoid maturation may not be linearly equivalent to in vivo retinal development. Therefore, some metabolic differences observed between day-28 MRO and P3 retina may reflect developmental asynchrony. In addition, GC–MS metabolomics measures relative metabolite abundance rather than metabolic flux or direct functional activity; moreover, several unknown signals were encountered that were not included to in-depth data analysis. Functional and flux-based studies will therefore be needed to further validate these findings.

GC–MS also has limited sensitivity for complex lipids, retinoids, and low-abundance signaling molecules, which may result in the underrepresentation of specific photoreceptor-associated metabolites. Targeted metabolomic analyses of these metabolite classes could provide a more comprehensive understanding of the metabolite abundance.

The intracellular metabolomic comparison was performed at a single developmental stage, although retinal metabolism is highly dynamic during maturation. Longitudinal profiling would help distinguish delayed maturation from intrinsic organoid-associated metabolic differences. In addition, temporal changes in conditioned-media metabolite profiles may reflect both organoid maturation and stage-specific changes in culture conditions, including supplementation with N2 and B27 neural culture supplements, retinoic acid, and taurine. These extracellular metabolite differences should therefore be interpreted as reflecting the combined influence of biological maturation and the culture environment.

Although the relatively small number of biological replicates limits the statistical power to detect individual metabolite differences, this study provides an exploratory metabolic characterization of mouse retinal organoids and establishes a GC–MS-based pipeline for comparison with mouse retina. Given the small sample size, multivariate analyses, particularly PLS-DA, should be interpreted as exploratory and require validation in larger independent datasets. The identified metabolic patterns provide a foundation for hypothesis generation and future validation in larger independent datasets.

Finally, retinal organoids lack several in vivo retinal features, including retinal pigment epithelium interaction and vascular-associated nutrient exchange, which may contribute or be a consequence to the metabolic differences observed between MROs and native retina.

## Conclusion

In conclusion, MROs present a substantially similar retinal metabolic signature while exhibiting selective metabolic divergence from mouse retina. Altered abundance of glycolysis-, TCA cycle-, lipid-, and neurotransmitter-associated metabolites suggests incomplete metabolic maturation under current in vitro culture conditions. Nevertheless, the substantial overlap between MROs and native retina supports the utility of MROs as retina-like metabolic systems. Identification of these metabolic differences provides a framework for future optimization strategies aimed at improving retinal organoid maturation and functional fidelity.

## Supplementary Information

Below is the link to the electronic supplementary material.

Supplementary file1 (DOCX 22 kb)

Supplementary file2 (CSV 35 kb)

Supplementary file3 (CSV 1 kb)

Supplementary file4 (CSV 8 kb)

Supplementary file5 (CSV 34 kb)

Supplementary file6 (DOCX 768 kb)

## Data Availability

The metabolomics and metadata reported in this paper are available in Radboud Data Repository https://doi.org/10.34973/ew8v-gp51.
