# Supplementary material for: Metabolic profiling of retinal organoids reveals conserved core metabolites and alterations in glycolytic and amino acid metabolism
Source: Metabolomics. 2026 Oct 10;22(6):171. doi: 10.1007/s11306-026-02523-4 (PMC13653828; doi:10.1007/s11306-026-02523-4)
Supplement: Supplementary file 1 [file 11306_2026_2523_MOESM1_ESM.docx]

**Metabolic Profiling of Retinal Organoids Reveals conserved core Metabolites and Alterations in Glycolytic, and Amino Acid Pathways**

Pragati Gupta^1,2*^, Claudia Wiesner^3*^, Fucke Zimmsen^2^, Jeroen Klevering^1^, Purva Kulkarini^4^, Zohreh Hosseinzadeh^1#^

1 Department of Ophthalmology Radboud University Medical Centre, Nijmegen, Netherland

2 Department of Ophthalmology and Eye Hospital, Leipzig University, Leipzig, Germany

3 Institute of Analytical Chemistry, Faculty of Chemistry, Leipzig University, Leipzig, Germany

4 Laboratory of Genome Diagnostics, Department of Human Genetics, Radboud University Medical Centre, Nijmegen, Netherlands

*shared authorship

#corresponding author

Zohreh Hosseinzadeh - Zohreh.hosseinzadeh@radboudumc.nl

**Supplementary table 1**

| **RD (Retinal Differentiation) Media (Day 0- Day 7)** | |
| --- | --- |
| **Component** | **100ml** |
| GMEM | 96.4ml |
| KSR | 1.5ml |
| Non-Essential Amino Acids | 1ml |
| Sodium Pyruvate | 1ml |
| Mercaptoethanol (0.1M) | 100μl |
| **Tom’s Media** | |
| **Component** | **100ml** |
| DMEM:F12 | 98ml |
| Non-Essential Amino Acids | 1ml |
| Sodium Pyruvate | 1ml |
| **From Day 7 to Day 15**: Toms media with addition of following supplements | |
| N2 (100x) | **1ml** |
| B27 (50x) | 2ml |
| **From Day 10 to Day 15**: Toms media with addition of following supplements | |
| Retinoic acid (1mM) | 0.5μM |
| Taurine (1M) | 1mM |
| **From Day 15 to Day 28**: **Toms media without any supplements** | |
| NOTE: Filter with 0.22 uM filter membrane before adding B27, N2, Taurine, and Retinoic acid | |

**Supplementary table 2**

Overview of primers used for Qpcr

| S. No. | Gene Name | Primer | Primer sequence (5'- 3') |
| --- | --- | --- | --- |
| 1 | Prkca | Forward | TTGTCCAAGGAAGCCGTCTC |
| 2 | Prkca | Reverse | CTCCTTTGCCACACACTTTGG |
| 3 | Actb | Forward | GCTCCGGCATGTGCAAAG |
| 4 | Actb | Reverse | CCCACCATCACACCCTGG |
| 5 | Opn1sw | Forward | GGCTCTGTAGCAGGTCTAGTG |
| 6 | Opn1sw | Reverse | CATGCTTGGAGTTGAAGCGG |
| 7 | Pax6 | Forward | CTGAGGAACCAGAGAAGACAGG |
| 8 | Pax6 | Reverse | CATGGAACCTGATGTGAAGGAGG |

**Supplementary table 3**

Program for GoTaq (SYBR Green) Qpcr Analysis

| Program Name | Cycles | Temperature(°C) | Time (mm:ss) |
| --- | --- | --- | --- |
| Hold Stage | 1 | 50 | 02:00 |
|  |  | 95 | 10:00 |
| PCR Stage | 45 | 95 | 00:15 |
|  |  | 60 | 01:00 |
| Melting Curve | 1 | 95 | 00:15 |
|  |  | 60 | 01:00 |
|  |  | 95 | 00:15 |
