# Supplementary material for: Metabolic profiling of retinal organoids reveals conserved core metabolites and alterations in glycolytic and amino acid metabolism
Source: Metabolomics. 2026 Oct 10;22(6):171. doi: 10.1007/s11306-026-02523-4 (PMC13653828; doi:10.1007/s11306-026-02523-4)
Supplement: Supplementary file 2 [file 11306_2026_2523_MOESM6_ESM.docx]

Metabolic profiling of retinal organoids reveals conserved core metabolites and alterated glycolytic, and amino acid metabolism

Pragati Gupta^1,2*^, Claudia Wiesner^3*^, Fucke Zimmsen^2^, Jeroen Klevering^1^, Purva Kulkarini^4^, Zohreh Hosseinzadeh^1#^

1 Department of Ophthalmology Radboud University Medical Centre, Nijmegen, Netherland

2 Department of Ophthalmology and Eye Hospital, Leipzig University, Leipzig, Germany

3 Institute of Analytical Chemistry, Faculty of Chemistry, Leipzig University, Leipzig, Germany

4 Laboratory of Genome Diagnostics, Department of Human Genetics, Radboud University Medical Centre, Nijmegen, Netherlands

*shared authorship

#corresponding author

Zohreh Hosseinzadeh - [Zohreh.hosseinzadeh@radboudumc.nl](mailto:Zohreh.hosseinzadeh@radboudumc.nl)

**Supplementary results**

**PLS-DA model validation of mouse retina and mouse retinal organoid (MRO) metabolomes**

Cross-validation analysis was performed to evaluate the robustness of the PLS-DA model generated from GCMS metabolomic data obtained from mouse retina and MRO (Supplementary Fig. S1). The optimal model was achieved using two latent components, producing a high explained variance (R² ≈ 0.99) and strong predictive ability (Q² ≈ 0.88). Model accuracy remained consistently high across component numbers. These results indicate that the observed separation between mouse retina and MRO is supported by a robust and predictive multivariate model with a low likelihood of substantial overfitting.

***Fig. S1:*** *Cross-validation analysis of the partial least squares discriminant analysis (PLS-DA) model generated from GCMS metabolomics data of mouse retina and MRO. Cross-validation was performed to assess model robustness and predictive performance and to reduce the risk of model overfitting. The obtained Q² and R² values indicate the predictive capability and explained variance of the PLS-DA model.*

**PLS-DA model validation of developmental stages of organoid media samples**

Cross-validation analysis demonstrated strong performance of the PLS-DA model generated from media collected across MRO maturation stages (Supplementary Fig. S2a). Cross-validation was performed to evaluate model robustness, predictive accuracy, and reliability of group separation while minimizing the risk of overfitting. Corresponding Q² and R² values represent the predictive performance and explained variance of the model. The optimal model was achieved using six latent components, producing high explained variance (R² = 0.99) and predictive ability (Q² = 0.84). These results indicate that the model captured a substantial proportion of the variance associated with developmental stage-dependent metabolic differences.

Permutation testing was performed to assess the statistical validity of supervised clustering and evaluate potential model overfitting (Supplementary Fig. S2b). The original model demonstrated superior predictive performance relative to permuted models, supporting the reliability of the observed metabolic separation between maturation stages.

The observed classification statistic (0.964) was greater than most values generated from 1,000 random permutations, yielding a permutation p-value of 0.064. Although slightly above the conventional significance threshold of 0.05, the result supports the presence of stage-dependent metabolic variation in conditioned media and suggests that the observed separation is unlikely to be driven solely by random class assignment.

***Fig. S2: Validation of PLS-DA Models for MRO Culture Media Across Different Maturation Stages.*** *(a) Cross-validation analysis of the partial least squares discriminant analysis (PLS-DA) model generated from GCMS metabolomics profiling of MRO culture media collected at different maturation stages. (b) Permutation test validation of the PLS-DA model derived from GCMS metabolomics analysis of MRO culture media across different maturation stages.*

**Metabolic changes in central carbon and neurotransmitters in MRO**

To further characterize alterations in central carbon and neurotransmitter metabolism, individual metabolites involved in glycolysis, the pentose phosphate pathway (PPP), the tricarboxylic acid (TCA) cycle, and neurotransmitter metabolism were examined. These metabolites are presented as representative boxplots in Fig. S3 using the same dataset shown in the heatmaps in Fig. 5 (statistical analyses in Supplementary Excel 3) . MRO exhibited reduced abundance of several glycolytic intermediates, including glucose-6-phosphate, fructose-6-phosphate, dihydroxyacetone phosphate, and pyruvic acid, compared with mouse retina (Fig. S3a). Similarly, PPP-associated metabolites, including ribulose-5-phosphate and myo-inositol-2-phosphate, were decreased in MRO (Fig. S3b). Several TCA cycle intermediates, including citric acid, fumaric acid, and malic acid, also showed lower abundance in MRO, whereas succinic acid was comparatively preserved (Fig. S3c). In addition, neurotransmitter-associated metabolites, including GABA and serotonin, were reduced in MRO, while glycine levels showed comparatively smaller differences between groups (Fig. S3d). These findings further support selective alterations in central carbon metabolism and neurotransmitter-associated pathways in MRO relative to native mouse retina.

***Fig. S3: Alterations in carbon and related neurotransmitters metabolism in MRO.*** (a-d) differential expression of key metabolites in mouse retinal (red) and retinal organoids(green).(*a) Glycolytic entry and upper glycolysis metabolites. Boxplots showing the relative abundance of glucose, glucose-6-phosphate, fructose-6-phosphate, dihydroxyacetone phosphate, and pyruvic acid. (b) Pentose phosphate pathway (PPP) and associated sugar phosphate metabolites. Boxplots illustrating changes in myo-inositol-2-phosphate, C6-6-phosphate, and ribulose-5-phosphate. (c) Tricarboxylic acid (TCA) cycle intermediates. Relative levels of succinic acid, citric acid, fumaric acid, and malic acid detected in the metabolomic analysis. (d) Neurotransmitter-associated metabolites. Distribution of glycine, 4-aminobutyric acid (GABA), and 5-hydroxy-tryptamine (serotonin). All metabolites shown were significantly different between groups (in Supplementary Excel 3).*

**Differential metabolite abundance between mouse retina and MROs.**

In Fig. S4, Volcano plot analysis demonstrated that the majority of detected metabolites showed comparable abundance between mouse retina and MRO, indicating broad conservation of the retinal metabolome (statical analyses in the *supplementary Excel Sheet 2*). A subset of metabolites was significantly altered between groups, with both increased and decreased abundances observed in MRO relative to mouse retina. These differentially abundant metabolites contributed to the metabolic separation observed in the multivariate analyses and were primarily associated with central carbon metabolism, amino acid metabolism, and lipid-associated pathways.

***Fig. S4. Volcano plot of differential metabolite abundance between mouse retina and MROs.*** *Each point represents a detected metabolite. Metabolites with significantly increased abundance in MROs are highlighted in red, whereas metabolites with significantly decreased abundance are shown in blue. Significantly different between groups could be found in Supplementary Excel Sheet 2.*

**Metabolites in MRO conditioned media across different developmental stages**

Analysis of conditioned media collected throughout MRO differentiation revealed dynamic changes in extracellular amino acid and neurotransmitter-related metabolites. Figure S5 presents representative boxplots of selected metabolites from the same dataset shown in the heatmaps (Fig. 6), illustrating their relative abundance across developmental stages (statistics analyses in supplementary Excel 4). Alanine, glutamine, glutamic acid, glycine, taurine, 4-aminobutyric acid (GABA), nicotinamide, and pyroglutamic acid displayed stage-dependent changes in relative abundance across the culture period (days 8–28). Several metabolites, including glutamate, glycine, and pyroglutamic acid, showed increased extracellular abundance at later developmental stages, indicating progressive metabolic remodeling during MRO maturation. These findings demonstrate temporal regulation of amino acid and neurotransmitter-associated metabolism in the MRO secretome.

***Fig. S5:***  ***Differential major metabolites in MRO* Secretome *at different days of collection.*** *Boxplot analysis revealed relative abundance changes of Alanine, Glutamine, 4-aminobutyric acid, Nicotinamide, Pyroglutamic acid, Glutamic acid, Taurine, and Glycine across different media time points (Day 8, 9, 10, 13, 14, 15, and 28).* *significantly different between groups could be found in Supplementary Excel Sheet 4.*

**Differential pairwise similarity correlation (DPSC) network analysis**

To investigate coordinated metabolite relationships beyond individual metabolite abundance changes, DPSC network analysis was performed for mouse retina versus MRO and across conditioned media collected during organoid maturation (Supplementary Fig. S6).

For the retina–organoid comparison (Supplementary Fig. S6a), the network consisted of interconnected metabolite nodes linked by both positive (orange) and negative (blue) differential correlations. Several metabolites formed local subnetworks connected through multiple positive and negative associations, indicating that differences between retina and organoids extended beyond individual metabolite abundance changes and involved alterations in metabolite correlation patterns. The network contained multiple branching structures and interconnected clusters distributed throughout the network.

For conditioned media collected during MRO development (Supplementary Fig. S6b), DPSC analysis also identified extensive positive and negative metabolite associations. The network displayed several connected metabolite clusters linked through shared correlation relationships. Multiple metabolites occupied central positions within the network and were connected to surrounding metabolites through both positive and negative correlations, indicating dynamic changes in coordinated metabolite relationships during organoid maturation.

Overall, DPSC analysis demonstrated that both intracellular retinal metabolomes and conditioned media metabolomes exhibit distinct metabolite association patterns, highlighting coordinated metabolic remodeling during MRO development.

***Fig. S6:***  ***DPSC network analysis in metabolites at tissue and Condition media in MRO.*** *(a) Differential Metabolite Network Organization Between Mouse Retina and MRO and (b) Differential Metabolite Network Organization across different days of organoid media collected during development. Each node represents a metabolite, edges represent pairwise similarity correlations between metabolites, red edges indicate a positive and blue edges a negative differential correlation and edge thickness corresponds to the magnitude of change.*
